# In their own words: disaster and emotion, suffering, and mental health

**DOI:** 10.1080/17482631.2018.1440108

**Published:** 2018-03-01

**Authors:** Ashleigh Elain McKinzie

**Affiliations:** ^a^ Department of Sociology and Anthropology, Middle Tennessee State University, Murfreesboro, TN, USA

**Keywords:** Emotion, mental health problems, disaster recovery, social suffering, trauma

## Abstract

**Purpose:** In this article, I explore emotions, trauma, and mental health issues residents experienced after tornadoes in Tuscaloosa, Alabama and Joplin, Missouri in 2011. **Methods:** The research is based on 162 interviews and fieldwork from 2013-2015. I draw from literature on social suffering and trauma to ask how experiencing mental health and trauma changes how people make sense of their social worlds. **Results:** I discuss four common themes: 1. Emotions in immediate aftermath, 2. Relationship strain, 3. Mental health problems, and 4. Emotions in long-term recovery.  Throughout the article, I pay attention to the bodily experiences of suffering and trauma. **Conclusion:** I argue experiencing mental health and suffering may be a critical perspective—one that can shed light on being in the world in ways that other perspectives may be less suitable to do.

## Introduction

In 2011, in Joplin, Missouri and Tuscaloosa, Alabama an EF5 and EF4, respectively, tore through each of these cities causing significant losses of life, property, and much suffering. That disaster engenders or exacerbates mental health problems has been well documented in the literature and is one the most cited examples of an event that can cause post-traumatic stress disorder (Davidson & McFarlane, 2006; Goldmann & Galea, 2014; Norris, Friedman, & Watson, 2002b; Norris et al., 2002a). In this article, I examine emotions, mental health problems, and suffering that were the result of these tornadoes. I draw on interviews with 162 people in both locations and fieldwork that spans from 2013–2015. My overall argument is that mental health problems, emotions, and suffering are something people experience *in their bodies*. In making this argument, I depart from most biological and social scientific accounts of mental health, as if it is just something “out there”. I tie the limited scholarship in the social sciences about what mental health problems *actual feel like* to scholarship on social suffering. Moreover, as Kleinman, Das, and Lock (1997) contend, suffering is produced by the social order. However, what makes suffering seem individualized, or something that could cause stigma, is due to the overall tendency in our culture and medicine to compartmentalize mental health and suffering as a personal problem. In this article, I ask: How are emotions, suffering, and mental health problems experienced after disaster and how do these experiences help residents make sense of their lives in the post-disaster landscape?

It is important to keep in mind that social suffering and emotions produced by weather events is in no way natural. This is the case for at least three reasons. First, existing inequalities or inequalities that are created by tornadoes are not inevitable—that is, they come from somewhere. Second, disastrous weather events are linked to human-made (and socially produced) climate change (McKibben, 2011). Though it is difficult to claim definitively any particular storm is caused by climate change, there is a correlation, and climate scientists are nearly unanimous in their predictions that disasters will rise in incidence and severity as human-induced climate change occurs. Third, though suffering is often thought to be private, it has very real effects on relationships and how people make meaning of their lives and their suffering.

## Literature review

In this literature review, I discuss subjective experiences with mental health, interdisciplinary treatments of social suffering, and the body and emotions. Sociologist David Karp’s (1996) book *Speaking of Sadness: Depression, Disconnection, and the Meanings of Illness* is an important starting point. While reading other scholarship (Aneshensel, Phelan, & Bierman, 2013; Johnson, Turner, & Link, 2014), I was repeatedly frustrated because many social scientists and some practitioners do not explicitly document the *subjective* experiences of mental health problems. Instead, most work tends to hold to positivistic interpretations and measurements of mental illness. Though there is the understanding that mental illness is socially constructed, the over-reliance on positivism to interpret and investigate mental illness is disappointing because it reifies the Cartesian mind/body split.

It is my hope that this article will add to current literature the bodily experience of mental health problems. It is not my wish to necessarily answer the questions of *who* experiences mental health problems and *why*, but, instead, to show the subjective experiences of mental health problems and how those experiences both help people make sense of their lives.

### Other approaches to suffering and mental health

Ian Wilkinson (2005) contends that suffering is a slippery concept because there are many definitions and, secondly, suffering often seems to be personal. Moreover, suffering is thought to be less objective than pain. Wilkinson writes, “in contrast to pain, the domain of suffering is held to extend beyond the bounds of mere bodily sensation so as to encompass our entire experience of personhood in body, mind, and spirit” (p. 21). As I will demonstrate, those with mental health problems often feel as if those problems have taken over their life.

As aforementioned, suffering is an embodied experience. Whether suffering is the result of tornadoes, discrimination, mental health problems, or physical pain, etc., it is something we experience *in our bodies*. Kleinman et al. (1997) similarly contend that suffering is ignored because it is thought to be a personal and not a societal problem. Indeed, they argue that our societal response to suffering is some sort of treatment that will ameliorate the problem instead of taking a broader view. They also call attention to the idea that our obsession with normality and abnormality “obscure greatly consequential workings of ‘power’ in social life” (p. xi). Kleinman and Kleinman (1997) argue that suffering is a feature of life and that everyone suffers at some point in their lives.

I am also grateful for Karp and Birk’s (2013) contention that mental health problems should be understood as a critical standpoint. They assert that though we are aware of the importance of understanding what things *mean* to people, this tends to get lost in some studies of mental health. They argue, “A static picture of ‘having’ mental illness does violence to the complexity of an ongoing, emergent experience. We need to be committed to methodological and theoretical approaches that satisfactorily convey the moving, processual, and context-bound nature of any illness experience” (p. 25). In a similar way to social suffering scholars, they argue that what is needed is to actually listen to people. They conceptualize this as “the politics of listening”. They draw from Guinier and Torres (2002) who liken the canary’s call to the miners to the voices of the subjugated. Those who are marginalized from a variety of social locations can call attention to processes that are often not heard, overlooked, or ignored, which is the point of standpoint theory in general. They also maintain “For the mentally ill, mental health is another dimension along which one can stand outside the mythical norm, and yet it is this very status as an outsider that gives the mentally ill a valuable perspective on the otherwise invisible norms propelling their marginalization” (p. 29).

This is where I take inspiration from Karp and Birk (2013). I present the rest of the information in the article in a way that is attentive to what experiencing emotion, mental health problems, suffering and other traumas *feels like*. I try to do so in a way that does not reify mental illness or wellness and is attentive to the bodily dimensions of sadness, phobias, and other problems in order to show how these experiences influenced how my participants made sense of their worlds in the post disaster landscape.

### Emotions and suffering in long-term recovery

I also draw from perspectives that focus on how the body is a site on which the social is written (Bordo, 2003; Sutton, 2010). The framework I use to understand these processes borrows from sociological phenomenological accounts of embodied emotion (Denzin, 2007; Katz, 1999). This intervention argues that both positivistic (Turner & Stets, 2005) and constructivist theories (Boiger & Mesquita, 2012; Holyfield, Cobb, Murray, & McKinzie, 2013) of emotion elide the bodily experience of emotion (and trauma). This approach argues for understanding the body as an extension of how we experience ourselves in the world. Moreover, understanding emotion as both something that is part of meaning making and arising from interaction obviates some the messier arguments within social science about what, exactly, emotion is. If, in my understanding, a participant understands something as emotional, then it is emotion. A focus on emotion as coming from and part of the body avoids some of these sticky and, at times, overlapping constructions.

## Methods

I made use of three methods: participant observation, interviews, and archival work. The interviews were semi-structured, I asked probing questions, and the interview guide was arranged thematically (Emerson, Fretz, & Shaw, 1995; Lofland, Snow, Anderson, & Lofland, 2006). The interview guide was arranged around four sub-themes of questions: 1. Experience with the tornado; 2. Emotional response; 3. Community and government response; and 4. Personal hardships. I asked 29 questions in total.

During my graduate school training in sociology and women’s studies, I did preliminary fieldwork and interviews in both Joplin and Tuscaloosa. Participants were recruited by a variety of methods. I used contacts I had in Joplin (my partner is from Joplin) to snowball sample. In Tuscaloosa, I relied on a key informant to help me identify people to interview. I also spent time as Mexican restaurants, Black churches, and spoke with professionals in both areas to gain access to participants of colour. I spent 4 months in both locations beginning in May of 2014. There were no exclusion criteria except for age (that is, I did not interview anyone under the age of 18). If people wanted to talk to me, I interviewed them. The only exception is when the research was coming to a close and I was purposefully only interviewing participants of colour to boost those numbers in my sample. I interviewed people who were directly affected by the tornado. However, I also interviewed local leaders and those who were involved in cleaning up the city. The leaders are also included in the 162 interviews. The dichotomy of affected vs. non-affected is somewhat false because only five people did not report being affected by the storm in some way. However, my reasoning for interviewing those were not *directly* affected was because I wanted to try to paint as holistic of a picture as I could. My total number of participants was 162 with roughly equal numbers of men and women, over 50 vs. under 50, and working class vs. middle class. In terms of race and ethnicity, my Joplin sample was close to 30% non-white and Latinx. In Tuscaloosa, my sample was 45% people of colour. I only did one interview per person, except a few life histories I additionally completed on people whose stories I thought might be helpful. The interviews were audio-recorded and I transcribed half of the interviews and used grant money to pay someone to transcribe the other half.

I also volunteered part time with disaster organizations. My work with disaster organizations included answering telephone calls and putting people in need with other people or organizations who had resources. Research collection was an iterative process. I analysed some data in the field and adjusted my interview guide and foci (Emerson et al., 1995; Lofland et al., 2006). I engaged in initial coding, wherein any themes that seemed important were recorded. In my second round of coding, more focused, I started to pick out themes and ideas that were recurrent or seemed to help to answer my research question, and that challenged my assumptions. Then, at the time of drafting my papers and book from the research, I engaged in intensive and extensive memo writing—some of which is part of the analysis presented here. I did all of the coding by hand in Microsoft Word.

I was influenced by both grounded theory and the extended case study method. To simplify a long debate in qualitative research, the former is a more inductive approach and the latter, deductive (Burawoy, 1998; Charmaz, 2014). I engaged in line-by-line coding looking for the key “*in vivo*” (participants’ words and world-view) concepts and codes. I also developed “small theories” and looked for relationships between the open codes (Charmaz, 2006). Second, I engaged in focused coding which is the process of identifying relationships between grouped open codes and families of codes. I searched for reoccurring themes, theoretical ideas, and methodological challenges. Some of the open codes consequently faded in importance and relevance. In these first two steps, I developed memos that explored theoretical ideas, hypotheses, and constantly compared these ideas and hypotheses to the data as I continued analyses. Third, and after completing the first two rounds of coding, I again read and referred back to existing empirical literature and theories about disaster and inequality to ascertain what I could contribute and how my research complicated existing frameworks and theories (Burawoy, 1998). Using insights from the literature, I engaged again in line-by-line, open coding for new insights, ideas, or negative cases that I might have missed during the first round of analysis.

My role was mostly as an observer and interviewer. I engaged in participant observation as much as possible but I did not have access to sites that would put me into direct contact with participants as they led their daily lives. Thus, I spent a great deal of time during the interviews trying to get to know my participants. Sometimes, I would spend several hours at their houses before or after the interview. In some instances, I became friends with my participants and spent subsequent time with them and in their lives. The interviews lasted from one to three hours.

My position as a working class white woman was on my mind during the entire research process. I had trouble gaining access to people of colour in both locations and learned to be very specific about my politics and what I was hoping to accomplish with my research. During my time in both locations, I endeavoured to spend as much time at town events as was possible, such as city council meetings and leisure events sponsored by each city. I wrote extensive field notes about my interviews, interactions, and events. I made sure to pay attention to findings or themes that challenged my understandings of long-term recovery. I obtained Institutional Review Board approval and the participants signed informed content. The informed consent procedure ensured that they could quit the process at any time. I was aware that talking about traumatic events could reinscribe suffering and tried to be as careful as I could to, say, let participants cry if they needed. Often times, I would cry too. Moreover, all names presented here are pseudonyms. No actual names are used. Methodological saturation was achieved long before I finished interviewing 162 people; however, there were so many folks who wanted to speak with me about their experiences that I felt obligated to comply with their wishes to be interviewed.

My position in the world is different from that of others. I went into the research setting with my own presuppositions and epistemological standpoint. Thus, in terms of reliability, I scrutinized my actions and decisions in a way that is equivalent to doing research with the thought that someone was looking over my shoulder. This allowed me to be able to explain to others what I did and why. I kept journals that detailed my ideas and decisions I made in the process of research for both reliability purposes and my overall commitment to ethics and reflexivity. Moreover, I argue that the question of truthfulness, or validity, can sometimes be misleading for qualitative researchers. Thus, I made every attempt to represent my participants in a way that did justice to the stories they told me and for the overall goal of improving people’s experience with disaster. I subscribe to the notion of transformational validity which recognizes multiple perspectives (mine included), emphasizes researcher reflexivity, positionality, and has the end goal of empowerment (Cho & Trent, 2006).

## Nightmare on my street: recovery written on the body and mind

Participants told me two, three and four years after the storm, mental health problems and negative emotional responses had started to get better; however, for others they did not. It is of little wonder, then, that a tornado would engender mental health problems and emotional discomfort. Compounding that situation of experiencing horror, surreal surroundings, and a loss of reality is the struggle to recover. I present my findings based on four common themes: 1. Emotions in the immediate aftermath; 2. How emotions and trauma often caused relationship strain; 3. Experiencing mental health problems; and 4. Experiencing emotions *throughout* long-term recovery.

### Emotions in the immediate aftermath: tornado shit and experiencing the tornado

Not surprisingly, people did not have to lose a loved one or belongings to feel loss. Participants described their experiences with the tornado as inducing shock, being surreal, and causing much emotional discomfort. Moreover, most of my participants described the aftermath as if a “bomb had gone off”. Many participants recounted experiences of waking up and not knowing where they were, being trapped in rubble, and holding onto their loved ones for dear life. Some described the experience as “time was speeding up” and yet others as “time standing still”.

Most of my participants vividly recount the day of either 27 April or 22 May. In fact, when I asked them to tell me about their experiences with the tornado, it often took up a quarter and sometimes half of the interview. The ability to remember traumatic events in clear detail, as if it happened yesterday, is a common phenomenon that psychologists link to emotional arousal (Cahill & McGaugh, 1998). Often, people will be able to recall small details, for example, what they were wearing, when the event occurred. My participants told me that their immediate emotions ranged from disbelief, sadness, shock, sorrow, fear, worry, and feeling disoriented (or lost). They also discuss how these emotions actually felt.

Silas Halk, a retired African American man in Tuscaloosa, recounted to me exactly what was going through his mind and what he was wearing in the aftermath of the tornado. The tornado sucked him out of his house through the chimney. He recollected that he stepped over dead bodies, something he had never even told his wife. He had yelled at some youngsters to get off their bikes and take shelter when he realized that the tornado was going to hit—only to see their lifeless bodies moments later. Others still, such as Theresa Richards, a working-class African American woman, described exactly how it looked in Joplin:

Franklin Tech [a technical college], on fire, and telephone poles in the street and just people were just—I think it had to be like just shock, because people were walking around just I mean, totally lost, just—it almost looked—It’s almost like you were just taking up and planted into like a new civilization. (July 2014)

This was indeed common among my participants, that is, that people were in shock, disoriented and just wandering. Shock and disorientation or being “totally lost”, though culturally mediated, are bodily sensations. Sheila and Randon Turner, a young white couple in Tuscaloosa, told me, “And we open the door, and the world was changed” (September 2014).

Consider this lengthy excerpt from Scott, a white man in his mid-thirties from Joplin. His narrative is not unusual in any way, but it is an example of what most of my participants narrated: exact details, feelings of terror and the physical display of raw emotion, such as tears or crying. I asked Scott to tell me about this experience with the tornado and he answered:

My grandma is in my bedroom looking out my bedroom window and it started getting real um dark outside and just raining and just all kinds of crap. And so, uh, I told grandma because we were looking out the window and like I said, houses are everywhere, trees are everywhere…but when the trees are touching the damn ground you know there is something wrong with it. I told grandma, I don’t freak out in storms…well I do now. I told grandma, take my daughter and go in the bathroom. And so, uh, as she was walking out of my bedroom, the first thing I remember was my ears popping from all the pressure that was in here…it didn’t register I don’t guess, but I noticed the pressure in my ears. So they went to the bathroom and I was coming down the hall…because I’ve always been when my dad was alive when I was kid we went out and watched it, and you know. The way the house is set up now is basically the way that it was but there is walls missing right here.

And so I turned the corner and started coming down and this window was about twice the size it is now. It was just a huge ass bay window. And uh, (crying), and uh this window blew in and that’s when I got scared, and so I went in and I got in the bathroom and at that time is when everything started happening. And the wind and all the crap and I started hearing glass break and I couldn’t get the door shut. And so uh, I sat on the floor and I put my foot on the toilet and I leaned back as far as I could and it finally latched and my daughter crawled over to me and I held her and I held her head to make sure it was covered and I said, “don’t look, don’t look.” She kept telling me, okay, okay. (Crying). She didn’t look, she kept her head buried in my chest, she was really good.

And so we are sitting there and I could feel the house moving. It was just amazing power. And I could feel it, and all of the sudden the roof just left. And I told grandma, I said, “the roof’s gone.” And about the time the roof left, the exhaust thing came down and cracked her in the head and so she kind of rolled off the toilet and sat on the floor with us and the uh, the wall that was separating us from basically the outside was gone. It was protecting us, the wall in the bathroom and I mean you could feel it just moving. And I remember, you know, I remember holding my daughter and looking up and looking at this stupid thing as it went across the house and after it kind of calmed down and picked back up again. And of course they are talking about the eye of the storm and the eye of the tornado and all that stuff.

And after it started coming down I remember looking down and seeing all the insulation and everything and the stupid stuff you know, I am thinking you know, my toothbrush…all this shit is just flying around the room. And after it was all over, well almost all over, I opened my eyes and I stood up. And at this time, when I gotten back from getting pizza, I went to my bedroom and took out my keys, my wallet, my cell phone, took off my shoes. So none of us had shoes on, none of us had shoes on. I mean grandma had her night gown on, my daughter had her nightgown on. And the funny thing is I still had the shorts on that I was wearing that night and my t-shirt. (Scott Mayes, June 2013).

Scott was able to remember exact details from the night of the tornado like it had just happened. Scott was also visibly emotional and cried several times while telling me about his experience. Here Scott shows the importance of talking to people about their actual experiences after traumatic events, such as tornadoes. His experiences powerfully show how it feels to be a tornado survivor and the emotions it can engender.

Another common narrative from my participants was to describe how everything looked after the tornado. In addition to describing their horrors with what their towns looked like, such as a war zone, participants also similarly described how the tornado ruined their belongings. Megan and Tom Crawley, a white couple in their sixties in Tuscaloosa, told me:

Megan: Everything had this tornado—

Tom: *Tornado shit*. That’s what we call it.

Megan: Okay, (laughs) I was trying to come up with another word.

Tom: Okay, it was just dirt—

Megan: Insulation.

Tom: Insulation, pieces of wood, splintered wood, glass.

Megan: It was like somebody just sprayed it on everything.

Tom: It was coating everything that you tried to get out [recover], so—I don’t know how, but something that would be completely sealed up—

Megan: Yeah. It would be inside.

This narrative of tornado goo, tornado shit, mud, debris, and mould is ubiquitous in my interview data. Indeed, I witnessed it also when I was in Joplin volunteering in 2011. Many of my participants also described that this goo or shit felt as if it was not only invading their belongings but also their psyche and their bodies. A disaster event stuck with my participants like goo or paste.

### Strain and relationship problems

Six of my participants also discussed their how their relationships ended because of the tornado. In this short section, I want to briefly show how something as traumatic as a tornado can be an impetus for a relationship to change and how that is related to emotion and mental health. Bianca May, a middle class white professional in Tuscaloosa, found her husband cheating on her in their dilapidated house after the storm hit. When I asked her about mental health, she told me:

Like you know, I did do some treatment for PTSD afterwards but as the therapist and I were talking about, it was hard to differentiate like where was the PTSD really from, was it the tornado, was it the aftermath of my marriage. Like all the crazy. So, talk about like, imagery. You know, it’s like, your house, your home, your marriage is falling about in your house that has just fallen apart. It was like, “my gosh” (September 2014).

Here we see the confluence of mental health, relationship strain, and trauma from the tornado resulting in ending a relationship. Others described their situation as surviving the tornado but not their marriage. Javier Lopez’s wife left him in the aftermath of the Joplin tornado and used their FEMA money. He described the situation as one in which he was taken advantage of because of his immigration status. Similarly, Cora Davies’ (a white woman in Joplin) husband took their insurance money and fled to California. Her name wasn’t on the mortgage note and she fell into depression after he left. She told me:

I did start an anti-depressant. I asked my doctor cause I’m thinking “why am I doing so badly, why am I doing…” I can help other people but not myself. I mean we can have a *head* knowledge about incredible things. I knew I need help, but I didn’t know what kind of help. I started to see a therapist. So I did try to seek help. (July 2014)

Cora’s situation shows both how relationships and trauma engendered by a disaster can coalesce into a situation that requires seeking help for mental issues. Similarly, Cassie Simpson, a young woman from Joplin, told me that because of her husband’s behaviour after the tornado, she knew she wanted a divorce. Cassie was in the process of thinking about wanting a divorce but her husband’s behaviour after the tornado pushed her in that direction. Cassie told me:

I finally got ahold of my ex-husband. And I was—he’s like “Oh my god”! He’s like “I’ve been so worried”! you know, “The kids”! And this and that—I was like, “Well, the kids are fine. I left a note for your parents”. He knew that. And so, he met me over there and that’s the day I decided I was going to go through with the divorce because the first thing he said to me is like, you know, “F-U-C-K,” you know, worried about the kids and like, and didn’t say anything about me and we’d only been separated for like two months. He’s just screaming at me, you know, freaking out on me like I had done it or something or done something wrong. I was like “You know they’re okay. You knew they were okay. But, yeah, thanks for caring”, you know? So I was like “Yeah”, that day, I was like “Yeah, I’m definitely going through this divorce”. So. You know. Lack of concern. Here I was worried sick about him, I thought he was, you know, something had happened to him, and it took me three hours to find out he was okay. (June 2014)

While most of my participants spoke about how their family was a great source of comfort, others discussed how the tornado caused great strife in their relationship. Taylor Frank, a middle-class African American woman in Tuscaloosa, and her husband also had many problems after the tornado, to the extent that her husband filed for divorce. Taylor told me:

My husband is a minister and he’s kind of quiet. He is local from Tuscaloosa and I am not so it’s kind of, I didn’t have the same kind of childhood memory loss that he did. But his mother, she is from Rosedale and still lives in the public housing project, it wasn’t but that’s what grew up around. And they wouldn’t let him leave work and when it was over, they let them leave, but he could only drive a certain distance. They parked the cars and had to walk. Well, he walked through Rosedale, before they put the bodies up. And my husband was okay. But Wednesday night, that Thursday and that Friday was at the church, and by Sunday, he had crashed and was in the bed in the foetal position and some of our friends had come and got us. We tried to stay in the house and then we tried to find hotels. I can’t remember what we did the first couple of nights. I just remember it was pitch dark. But then that same year, he filed for divorce, we aren’t divorced now obviously, but he just really bugged out.

Taylor’s story about her husband shows how his emotional reaction to the storm affected their marriage and almost caused a divorce. In the same interview, she told me that she was certain her husband had PTSD.

The findings in this section show how an event like disaster and disaster aftermath can be a catalyst for change. Not only do the excerpts in this section show trauma and suffering, but also how those experiences can lead to relationship strain and sometimes can cause a relationship to end. Moreover, there is great attention to the body in these excerpts, from participants describing themselves yelling loudly to curling up in a foetal position. Moreover, as Cora put it, you can have a “head knowledge” about something, but feeling and experiencing emotion are much different than knowing about it.

### Mental health problems

In this section, I discuss mental health problems and begin with depression, anxiety/panic attacks, and PTSD. Though things like PTSD have measurable negative health effects, I let my participants define their mental health problems as they understood them. The most common response to the tornadoes was panic and anxiety.^1^ Some of my respondents had peri-panic attacks (while the event was happening) and almost all of them described their panic and anxiety as something that would happen when there was the threat of bad weather and/or if they had to drive into areas of town that were not yet repaired. I asked Jalessa McDonald, a young black woman in Joplin:

Me: Ok. So you mentioned this a little bit with your son, but how do you feel now when you hear sirens?

Jalessa: Oh god. Panic.

Me: Panic? Have you talked to someone about the panic and anxiety?

Jalessa: I have. Well, yeah, I have, but I can tell it’s kinda getting a little worse so I need, I’ve been meaning to…I need to go talk to someone about that because it has gotten a whole lot worse for me. I think I need [to] up [the medicine]. *laughs* I need it up and I need some more. I mean, my anxiety is up, I mean, through the roof to where I can’t even drive home on the highway. You know, I have to go the back way. I mean, there’s times when I’ve stopped on the freeway, just stopped in the middle of the road. (June 2014)

In a different way, Sharon Yokum, a retired white woman in Tuscaloosa, was worried about construction that was going to take place in Tuscaloosa. She told me:

Sharon: Yeah. Well, yeah, but I’m telling you, that’s giving me panic attacks. Because that—I’m getting anxiety over that. Once that bridge comes down, I’m going to get a panic attack over it, because I have no escape. Yeah, I can take that road, and I can take that road, and I can take that road, but the main road is going to be totally cut off, and I’ll be—I’m already panicking at the thought of it. I have panic attacks, and I’m just pacing the house, and I’m hyperventilating, and I’m nauseous and I’m losing it. (September 2014)

Doris Callahan a white lawyer in Tuscaloosa, who didn’t suffer long-term mental health problems, nonetheless had a panic attack and it scared her. She was with her son and had ventured out of her neighbourhood. She told me:

And so by the third day, [my husband] said I need those chainsaw chains. See if you—see if you can, you know, go down there and get them. So I knew about 15th Street, which is how you would go to go there, and I thought, okay, what I’ll do is I’ll go out Skyline and then I’ll go up Skyline to the end of Greensboro and drive up Greensboro that way, and that way I’ll miss the whole Forest Lake—you know, I won’t—you know, we didn’t have news, we didn’t have power still. I didn’t know about Rosedale. And I got—and [my son] was with me, and I got up to 10th Avenue on Greensboro, and you really couldn’t see much, but they wouldn’t let us go any further, and so I had to turn out over the interstate to turn around and come back, and when I got on that overpass, I could see just those metal warehouses up there that were blown down, and I had my panic attack. It’s the damnedest thing. It was completely out of my control. And I have always said that is bullshit. You know, just somebody talking about they had a panic attack. Give me a break. Well (laughs)—I just started crying and hyperventilating and I mean, my heart was pounding and [my son] was like, momma, it’s okay. It’s okay. They can build it back. (laughs) It was—But basically it was—I don’t know if it was a panic attack or a flashback, you know, or what. But—so I don’t poo-poo people who say they have those that much anymore. (October 2014)

Similarly, Joyce Rodgers in Joplin told me that her husband had to take time off work to deal with his panic and anxiety. (Many of my participants conflated panic and anxiety so I do not separate them.)

Depression was less commonly articulated, but many participants did mention having depression because of the storm. Alaina Pederson, a young black woman in Joplin told me, “Of course I ended up getting on like little anti-depression medicine or whatever, trying to keep me level, because there would be times you’re sitting in bed or you’re laying in bed with your family, and you’re just like ‘how many people can’t hug their kids now’” (July 2014). Alaina shows that stories of death and destruction weigh on people. Similarly, Magdelena Myers (who later talks about PTSD) also told me that she “fell into a deep depression” after the tornado (July 2014). Claire and Benjamin, a young couple in Joplin, talked about how they had to both start taking medication. Their experience was quite devastating. Claire was pregnant and near her due date when she rode out the tornado in their basement. Claire, who did most of the talking, told me:

Both of us are on anti-depressant and anxiety medicine because of it. It’s just because there for a little while it was November after the storm, or September, November, somewhere in that range, that I had wanted everything to be back to normal. Well, you would think April, September…You think that things would be completely fixed by then, and that was not the case, and I had a couple of panic attacks like where I really like couldn’t breathe, and then mostly though was just couldn’t get up, couldn’t get motivated, just didn’t want—Couldn’t deal. (June 2014).

Claire and Benjamin had a harrowing time with trying to get their house rebuilt. Claire, in particular, had anxiety, panic, depression, and PTSD.

Post-traumatic stress syndrome, or PTSD, usually refers to reliving a traumatic event, and disaster is one of the heralded causes of PTSD. Magdelena Myers, who almost died and lost close friends, narrated her experiences to me. While very few people disclosed having PTSD to me, the mental health professionals with whom I spoke, estimated that many people probably did have PTSD. Magdelena told me:

Magdelena: Yeah, right after the tornado everything—Oh man, my life fell apart. Their dad left me for another woman. They [her children] were not around anymore. And oh gosh, I ended up in just horrible, nasty rock bottom, because I thought I’m supposed to be dead. What am I doing here? So yeah, the first couple of months after the tornado I was grateful. I was like, thank you God for saving my life. I feel very empowered and I feel very grateful, but then I was upset. I felt like I didn’t deserve to live so I started to do anything possible to hurt myself. I had called the cops and told them to come and kill me because I was like help me, help me. When they come over I said “do you guys think you’ll help me by killing me and just act like it’s a crime scene, because I’m supposed to be dead from that tornado”. I went crazy on the cops. “Please, just help me, take my life away. I’m not supposed to be here”. And I was like that for months. “I’m not supposed to be here”. I was just hysterical. “I’m supposed to be dead”. I was suicidal. But I couldn’t do it. I wanted to kill myself. I was like how do I do this? I was scared and I wanted to die, but I was also scared of—I don’t know how to explain it, so many mixed feelings. It all hit me. It got very ugly. Then I wasn’t working. I went hungry a lot, and like I said, the kids were not around, my family members were not around. I just wanted to die. I was just a walking crushed spirit. They [the kids] were with their grandma and their dad. Living in Joplin still, and I ended up hanging out with the wrong crowd, all the wrong kind of people, trouble makers and my house became a flop house, so we all just got together and just had all kinds of strangers, and so one day I ended up in jail and when I was in jail I didn’t want to hurt anymore.

Everything is better now, thank God. And I do struggle. Like fireworks scare me now, because I think it’s the brain injury or—I don’t remember what happened, but like 4th of July we were at a fireworks show, and I was literally crying and it was too loud. I think it brings back memories. I have post-traumatic disorder now, I believe it was the storm, so I bet it’s that. Because before 4th of July nothing scared me. (laughs) And now my boss at work, I’m very jumpy, and I wasn’t like that before May 22nd. Like he has to come around very gently, “Magdelena I’m right behind you. Magdelena, I’m beside you”, and I’m like “Ah”! (laughs) I’m like “no”, but I can’t help it. If anything is behind me I tend to fear that—and all of a sudden I get feelings like somebody is going to kill me, like somebody is going to sneak up from behind me and I get those freaked out moments where I feel like anything is going to just pop out of nowhere and kill me. (laughs) I guess from the trauma.

Magdelena had previously told me that she has PTSD but we see from this narrative that she suffered from survivor guilt, she was suicidal, and was in total poverty. Moreover, Magdelena draws attention to the bodily elements of her suffering. Her experiences of feeling crazy, freaked out, hysterical and suicidal are not things that can be separated from her body. Indeed, the feelings she got when there were fireworks explicitly show how this suffering is embodied and can be relived and re-experienced.

In this section, I have shown how people feel mental health in their bodies. Their words and experiences show that we cannot divorce what happens in people’s minds with what happens in their bodies. This is important because we know very little about how experiencing mental health problems actually feels. Moreover, from a societal standpoint, it is important because it connects individual experiences with larger structural forces, such as disaster and climate change.

### Emotions in long-term recovery

While almost everyone in both locations experienced some of the same emotions in the immediate aftermath, for many, these emotions carried over to how they made sense of themselves several years after the tornado.Sally Morganson, an older black woman from Joplin told me that it still tugs at her heart to drive by areas of town that were destroyed.  Gloria Sharpe, from Tuscaloosa, also an older black women, describes being heartbroken. Tiffany Withers, a working class black woman, describes her experiences as being in fear. She told me, “I was *afraid* to come to Alberta City [a community in Tuscaloosa] for a long time. I didn’t come to Alberta City until the Habitat [an organization that helped her to rebuild her home] experience. That’s when I came to Alberta City, because I just didn’t want to come over here. I didn’t want to see it” (October 2014). Tiffany was describing fear over actually seeing the damaged landscape. Sharon Yokum also described being in fear three years after the Tuscaloosa tornado. She said:

I mean, it physically changes the landscape but it also changes the landscape of a life, because of the landscape changes and life changes and the life altering, and the fears, and everything else. Life is a landscape just like outside…I’m hyper vigilant now, but any kind of—There’s a lot of fear. There’s a lot of fear of losing other people, like the last time it came through it hit the kids. (September 2014)

Sharon was bi-polar and compellingly parallels the physical landscape with emotional, lived, or embodied landscapes. That is, when the physical world changes, it is something my participants felt in their bodies and experienced with great suffering and trauma.

Mervin, a black man in Joplin, and Dalton, a black man in Tuscaloosa, discuss how three years after the storm, they still cry about it. For Mervin, he cries over loss of life; Dalton, on the other hand, almost lost his life and when he didn’t, he ended up saving other peoples’ lives. He said that when he thinks about it, three years later, he is still quite emotional. Mervin’s uncle passed away protecting others. Dalton, similar to Scott earlier, described his emotions as a problem, “my problem is when I talk about it, I get very emotional” (November 2014).

For others, the emotion that continued into the long-term was guilt over surviving while others lost their lives and their belongings. Samuel, a young white man in Joplin, told me, “I am just thankful now to be alive. I could have easily died. It definitely weighs on you” (May 2013). James Lively similarly told me, “Now, that’s one thing. I mean, you do have some survivor’s guilt when you sitting there looking at your neighbour’s house and you’re like, I got a little roof damage and that guy’s got nothing” (October 2014). Sophia Carter, an upper-class professional in Joplin told me, “I felt guilty every day for, I don’t know, how many weeks that my lilies were blooming in my yard. I felt guilty, I mean, guilty every day. I didn’t know what it was. One day I remember saying to myself (laughs) ‘oh, this is what survivor’s guilt looks like’. Because we lost so much” (July 2014).

Others described long-term recovery as a *new normal* or that they would never actually return to normal due to their experience with the tornado. For others, they told me that the trauma from the tornado interrupted their sleep. Alexandria Chavez, a young Mexican woman, told me:

I didn’t sleep that night, I had nightmares…I had nightmares for actually months after that. Tornado nightmares, they would last two hours or so. Emotionally, like not there, I am not saying I was delirious but I was so very sad. I mean you hear, kept hearing like three days after of people dying or missing and your house is not there anymore and your car is messed up and not there anymore. So it was very overwhelming and very sad.

The Petersens, a black family from Joplin, conveyed to me that for all of them, sleep is elusive. Jalessa McDonald described that after the tornado “life after that was so different. It was like living, but not living” (June 2014). Some described the tornado and recovery as “making them sick to their stomachs”. Others still had flashbacks. These folks didn’t necessarily categorize their experiences as PTSD so I include them in my emotion category. A woman who had survived Katrina only to also survive the Tuscaloosa tornado told me:

[It was like] a sense of like loneliness, it reminded me of what happened back home. Like, okay, “what are we going to do? Is the people okay?” I remember a lot was lost back home and that was like, it made me like have a flashback and I was kind of like depressed for a very long time (Betsy Seiger, November 2014).

Claire Martin, a young white mother in Joplin, told me that she has flashbacks every Monday morning when they sound the test alarms. In short, my participants describe their emotions in ways that should not be divorced from their bodies. They feel their emotions in their bodies and experienced the tornado in their bodies—not as if it was just an experience “out there” that happened. Moreover, these emotional responses occurred two to five years after the storms and changed the ways in which they interacted with their social worlds.

## Discussion

In this article, I asked how emotions, suffering, and mental health problems are experienced after disaster, and how these experiences help residents make sense of their lives in the post-disaster landscape. In answering these questions I have shown how tornadoes caused emotions in the immediate aftermath and in long-term recovery. I have also shown how a traumatic event like a tornado can engender mental health problems and cause relationship strain or change.

Furthermore, several participants articulated explicitly how the tornado and long-term recovery changed the way they lived their lives and made sense of their lives. Joyce, a young biracial grandmother in Joplin, described being confused with what is and what is not reality, “I mean, just the devastation out there, and then it was like forever you drove down streets you’ve driven down for years, and you’re lost. You don’t even know where you’re at because nothing is the same” (June 2014). Similarly, Natalie Peden told me, “But if I would drive through a part of town that I normally didn’t drive through and see all the things that were gone, it was like a gut punch all over again. You know, it was like, oh, you know, wow—all the things that you expect to see aren’t there” (October 2014). Sometimes, people would avoid particular places because of the emotional reaction that it caused. For example, Carissa Foster, an African American woman in Joplin, told me:

I didn’t even drive down that street. I didn’t even go down 20th Street. And you got lost anyway, because there was no signs, but I just didn’t want to go in that area. I just got to the point where we stayed on the east side of town, and I was okay with that. I didn’t want to come—I even hated to drive to work, because I had to drive through it. Everything was gone, yeah. And you were just like—I think you’re just overwhelmed. And to drive through it when I did, I’m like ugh, it was—If you could close your eyes and drive through it, I guess it would be better, but you can’t. (July 2014)

Some participants couldn’t watch television about weather events. Claire Martin laughingly told me that she couldn’t even watch *Sharknado*. Additionally, Bianca May, a middle-aged professional from Tuscaloosa, told me that she used to feel excitement when there was a forecast for bad weather. She told me that she thought to herself, “is it coming here”? with a bit of glee. She no longer has the same excitement. Her house was devastated and the historic district, that is, a older neighborhood designated as historical, was razed of any foliage and instead of being a verdant, older neighbourhood, it felt desolate.

In short, for some it affected where they would and wouldn’t drive; for others, what they could and couldn’t watch on television. Lucia Perez said that her grades went down and many participants described the tornado as making them more aware. Emotion from the tornado took many forms and for most, it lasted two, three and four years after the storm.

In this paper, I have shown how trauma and emotion are experienced in the long-term for many people. Most work on mental health and suffering engendered by an event such as a disaster tends to look only at the short term. While many people do tend to get better, for others, the experience and pain sticks with them.

## Conclusion

In this article, I have argued for understanding the subjective experiences of mental health and emotion, specifically in the context of disaster recovery and disaster aftermath. This is important because it shows how residents understood their social worlds after and while experiencing trauma. Moreover, giving voice to their experiences is an important addition to literature that tends to hold to objective measures of mental health and quantitative methodology. I have also described experiencing mental health and emotions in a way that does not rely on positivistic measurements that can reify what experiencing suffering feels like in peoples’ bodies and what it can do to their lives. Additionally, I have pointed to the idea of understanding those who have mental health problems as having a particular type of perspective that is not available to others who do not suffer from the same problems. Indeed, if experiencing mental health problems gives insight to a person that is not obtainable to people without problems, then it can be understood as a way of seeing the world not available to everyone else. This is useful because it lends insight to understand suffering that is often marginalized and invisible. I suggest that future work should explore the subjective and interpretive experiences of mental health problems and emotions and should conceptualize mental health problems as a critical perspective. Moreover, future work should continue to investigate disasters and their aftermaths. As others have argued (Merton, 1969), disaster is a crisis event that can lay bare social structure in a way that settled times may be less suitable to do.

## Implications

Based on the overwhelming amount of criticisms of federal government articulated to me by my participants, I offer several policy suggestions that could help alleviate social suffering in the aftermath of disaster. First, there must be more funding for the aftermath of disasters. Our changing climate will cause disasters to happen more often and to be more severe. Disaster aftermaths and the changing climate obviously need to be a part of public policy in our society.

Second, rebuilding after disaster should be done in a way that takes people and the environment into account. We must, as a society, figure out some way to rebuild homes and businesses in sustainable ways. People in their local environments and our environment must be the chief concern when it comes to rebuilding—not the economy or making a dollar. Third, part of the increase in funding for disaster aftermath should directly deal with the mental health needs of people. Mental health funding currently does exist, but that so many of my participants told me I was the first stranger with whom they had shared their experience was startling. Some folks narrated feeling that mental health services wouldn’t really help them, others pointed to the stigma they anticipated they might feel if they “admitted they had a problem,” and some folks simply didn’t know that mental health services were available to them. Indeed, that many participants tearfully thanked me for hearing their story and endeavouring to tell their story seems to also point to the need for more mental health services in disaster aftermaths. Fourth, and finally, military and para-military organizations tasked to deal with disaster must have sensitivity training about race, ethnicity, and poverty. So many of my participants felt the National Guard was discriminatory and rude to them. Military personnel must have more cultural sensitivity training that ensures they don’t reinscribe trauma and fear on already vulnerable and traumatized citizens.

## Note

1. All the official definitions for these mental health problems can be found in the Diagnostic Statistical Manual-5.
